# Liposome-Encapsulated Rutin Attenuates Cisplatin-Induced Ototoxicity via Suppression of P53-Associated Oxidative Injury

**DOI:** 10.34133/bmr.0324

**Published:** 2026-02-10

**Authors:** Bo Liu, Yaqin Tu, Xiangrui Li, Wenting Yu, Wenqing Zou, Wei Tang, Shimin Zong, Songwei Tan, Hongjun Xiao

**Affiliations:** ^1^ Department of Otorhinolaryngology-Head and Neck Surgery, Union Hospital, Tongji Medical College, Huazhong University of Science and Technology, Wuhan 430022, China.; ^2^ Institute of Otorhinolaryngology-Head and Neck Surgery, Tongji Medical College, Huazhong University of Science and Technology, Wuhan 430022, China.; ^3^ Hubei Province Clinical Research Center for Deafness and Vertigo, Wuhan 430022, China.; ^4^ School of Pharmacy, Tongji Medical College, Huazhong University of Science and Technology, Wuhan 430030, China.

## Abstract

Cisplatin (CDDP) is a widely used chemotherapeutic agent, but its clinical applications are constrained by ototoxic side effects. Currently, few effective strategies exist to prevent or mitigate CDDP-induced ototoxicity. Rutin is known for its cell-protective effects by reducing oxidative stress and inhibiting apoptosis. However, its limited water solubility and inefficient delivery to the inner ear pose substantial challenges. To address this, rutin is encapsulated in liposomes (Lip-Rutin) for nanoscale drug delivery, leveraging its antioxidant properties. Lip-Rutin markedly attenuates CDDP-induced oxidative stress damage and apoptosis, demonstrating a protective effect on OC-1 cells. The efficacy of Lip-Rutin in safeguarding against CDDP-induced ototoxicity is further validated through in vivo studies. Consequently, Lip-Rutin emerges as a promising novel therapeutic agent for combating CDDP-induced ototoxicity.

## Introduction

Cisplatin (CDDP), a well-established chemotherapeutic agent, is frequently employed in clinical practice [1] for a range of solid tumors, including testicular, bladder, lung, head and neck, and ovarian cancers [2]. However, CDDP usage can lead to several adverse reactions, notably ototoxicity, nephrotoxicity, and neurotoxicity [1,3,4]. Reports indicate a high incidence of ototoxicity, with 40% to 60% of patients experiencing varying degrees of hearing loss post-treatment [5]. Currently, no clinically effective treatments exist for CDDP-related ototoxicity [6]. Contemporary clinical interventions for hearing impairment, such as systemic pharmacotherapy, intratympanic steroid administration, hearing aids, and cochlear implants, exhibit substantial limitations [7,8]. Systemic drug delivery is hindered by inadequate penetration of the blood–labyrinth barrier (BLB), necessitating elevated dosages that often fail to achieve therapeutic concentrations within the inner ear and provoke adverse systemic effects, including hypertension, hyperglycemia, and immunosuppression [9]. Although intratympanic injections enhance local drug availability, they remain constrained by the absence of sustained and targeted release mechanisms. These limitations underscore the imperative for the development of advanced drug delivery systems [10]. Nanoparticles (NPs), ranging from 1 to 1,000 nm in size, represent a promising alternative by prolonging drug retention and facilitating precise targeting within the cochlea, thereby potentially surmounting the drawbacks of conventional treatments while reducing systemic toxicity [11,12]. The mechanism underlying CDDP-induced ototoxicity remains unclear, though it is widely attributed to the production of reactive oxygen species (ROS) in the inner ear [13,14]. In recent years, researchers have developed various strategies to mitigate ototoxicity, focusing on antioxidant activity and antiapoptotic effects [13–15]. However, the effectiveness of these treatments remains limited, underscoring the ongoing need for effective interventions to manage CDDP-induced ototoxicity.

**Fig. 1.**
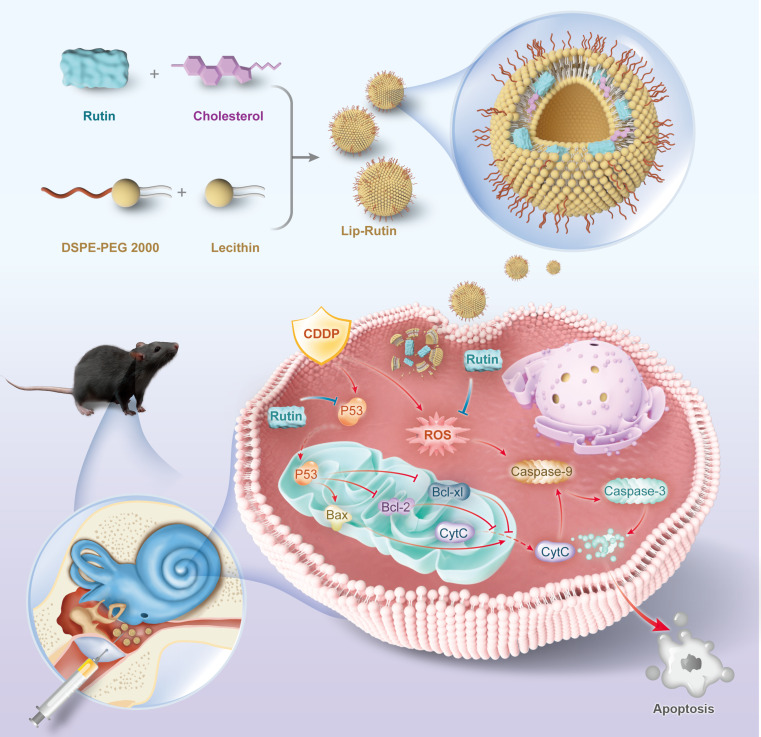
Synthesis of Lip-Rutin and its mechanism in resisting cisplatin ototoxicity.

Rutin, also known as quercetin-3-O-rutinoside and vitamin P, is a flavonoid compound renowned for its potent antioxidant properties, enabling it to neutralize free radicals effectively [16,17]. It has been associated with numerous biological effects, including neuroprotective [18], nephroprotective [19], antidiabetic [20], and anticancer properties [21]. However, due to its poor water solubility, low bioavailability, and difficulty in traversing the biological barriers of the inner ear, rutin is rarely utilized for inner ear disease treatment [16]. Studies have demonstrated that liposomes can transport drugs across the round window membrane into the inner ear, delivering therapeutic agents to inner ear hair cells (HCs) [22–24]. In this study, liposomes encapsulating rutin were created as Lip-Rutin to enhance its lipophilicity. The protective properties of Lip-Rutin against CDDP-induced ototoxicity were evaluated in various models, including the OC-1 cell line, Corti explants, zebrafish, and mice, along with molecular mechanisms responsible for these effects (Fig. 1).

## Materials and Methods

### Materials

Rutin, CDDP, cholesterol, lecithin, coumarin 6, and DSPE-PEG 2000 were procured from Aladdin (China). Trichloromethane, dimethyl sulfoxide (DMSO), and methanol were supplied by Sinopharm Chemical (China). Phosphate-buffered saline (PBS), fetal bovine serum (FBS), 0.25% trypsin, high-glucose Dulbecco’s modified eagle medium (DMEM), and DMEM/F12 medium were obtained from Gibco (USA). P53 and Bcl-2 antibodies were sourced from MedChemExpress (China). Bax antibodies were purchased from Immunoway (USA). CytC, caspase-9, cleaved caspase-3, Bcl-xl, GAPDH, and β-actin antibodies were acquired from Proteintech (USA). E3 embryo medium and 0.4% tricalcaine were purchased from Chuangxin (China).

### Preparation of Lip-Rutin

Lip-Rutin was synthesized using the thin-film hydration technique [25]. The process involved dissolving lecithin, DSPE-PEG 2000, cholesterol, and rutin in chloroform, followed by chloroform evaporation to form a lipid film. This film was then dried, hydrated with PBS, and centrifuged to collect the supernatant, resulting in the formation of Lip-Rutin.

### Characterization of Lip-Rutin

The particle size, zeta potential, and polydispersity index (PDI) of Lip-Rutin were assessed using dynamic light scattering (DLS) (Malvern Zetasizer Nano ZS90). Morphological examination of Lip-Rutin was conducted with a Talos F200X field emission transmission electron microscope (TEM) (Talos F200X, Netherlands). In vitro release of Lip-Rutin was evaluated using dialysis membranes with PBS as the release medium.

Stability of Lip-Rutin in high-glucose DMEM and PBS at 37 °C was monitored for 7 days. Fourier transform infrared spectroscopy (FTIR) (Bruker VERTEX 70v) characterized the NPs. The radical scavenging properties of Lip-Rutin were determined using the 2,2-diphenyl-1-picrylhydrazyl (DPPH) technique [26,27]. Antioxidant activity was quantified by calculating the percentage inhibition of the DPPH radical according to Eq. 1:

$$\text{Inhibition of DPPH}=\frac{\left(A_{0}-A_{n}\right)}{A_{0}}\times 100\%$$ (1)

*A*_0_ represents the absorbance values of the blank samples, and *A*_n_ represents the absorbance values of the mixed solution of Lip-Rutin and DPPH.

### Cell culture

In cell culture experiments, the immortalized HC-like OC-1 cell line, derived from the rat organ of Corti, was employed [28]. The cells were cultured in high-glucose DMEM medium supplemented with 10% FBS, maintained at 37 °C in a 5% CO_2_ incubator.

### Cellular uptake by OC1 cells

To investigate liposome absorption by OC-1 cells, Lip-Rutin-C6 was synthesized. OC-1 cells were seeded in 6-well plates at a density of 3.0 × 10^5^ cells per well and allowed to adhere overnight. Subsequently, the cells were exposed to Lip-Rutin-C6 for varying durations (0.5, 1, 2, 4, 8, and 12 h). After exposure, the medium was removed, wells were rinsed with cold PBS, and cells were fixed with 4% paraformaldehyde for 20 min. The cells were then stained with 4′,6-diamidino-2-phenylindole (DAPI) (Biosharp, China, BL105A) for 5 min. Liposome uptake was analyzed using fluorescence microscopy (Olympus, Tokyo, Japan) and flow cytometry (BD FACSCalibur, USA).

### Cell viability

OC-1 cells were seeded in 96-well plates at a density of 5.0 × 10^3^ cells per well. Various concentrations of CDDP and Lip-Rutin were added, and cell viability was assessed using a Cell Counting Kit-8 (CCK-8) assay after 24 h to evaluate the protective effect of Lip-Rutin against CDDP-induced cytotoxicity.

### Detection of ROS levels and mitochondrial membrane potential

Intracellular ROS levels in OC-1 cells were quantified using dihydroethidium (DHE) (MedChemExpress, China, HY-D0079). The mitochondrial membrane potential (MMP) was assessed with a JC-1 fluorescent probe (MedChemExpress, China, HY-K0601). OC-1 cells were cultured in 24-well plates and treated with either 10 μM CDDP or 50 μM Lip-Rutin prior to CDDP exposure. Subsequently, live OC-1 cells were incubated with DHE or JC-1 working solutions. Cell nuclei were visualized using Hoechst staining. ROS and MMP imaging were observed using a confocal laser microscope.

### Cell apoptosis analysis

Cell apoptosis was evaluated with the Annexin V-AbFluorTM 488/PI Apoptosis Detection Kit (Abbkine, China, KTA0002). OC-1 cells were harvested using trypsin, and cell precipitates were washed with cold PBS and resuspended in a binding buffer. Single-cell suspensions were stained with Annexin V-AbFluorTM 488 and PI, then analyzed by flow cytometry to determine the proportion of apoptotic OC-1 cells.

### Western blotting assay

Protein extracts were separated on a 10% SDS-PAGE gel and subsequently transferred to PVDF membranes. These membranes were blocked, incubated with primary antibodies overnight at 4 °C, washed with TBST, and exposed to a horseradish peroxidase-conjugated secondary antibody for 1 h at room temperature. Protein bands were visualized using a UVP BioSpectrum Imaging System.

### Cochlear explant culture

Cochlear explants from postnatal day 3 to 5 (P3 to P5) C57 mice were cultured in DMEM/F12 supplemented with 8% FBS. The cultures were maintained at 37 °C with 5% CO_2_ in a thermostatic incubator. HCs were damaged in vitro by incubating the cultures with 12 μM CDDP for 24 h. For the Lip-Rutin treatment, cochlear explants were pretreated with 50 μM Lip-Rutin 4 h before CDDP exposure.

### Immunofluorescence staining and HC counting

Cochlear samples were fixed with 4% paraformaldehyde for 1 h, then blocked with donkey serum and Triton X-100 for 1 h. Primary antibodies were applied overnight at 4 °C, followed by secondary antibodies for 1 h. Tissues were stained with DAPI and phalloidin for visualization, and HCs were quantified using a confocal microscope. ROS generation was assessed by staining with MitoSOX Red for 30 min, followed by DAPI and phalloidin staining, and observed using confocal microscopy.

### Evaluation of HC damage on zebrafish

Zebrafish larvae, aged 3 to 5 days post-fertilization, were obtained from Chuangxin, Wuhan, China, and cultured at 28 °C in E3 embryo medium with specific salt concentrations buffered with 4-(2-hydroxyethyl)-1-piperazineethanesulfonic acid (HEPES) at pH 7.2. The larvae were exposed to various concentrations of CDDP for 12 h, stained, and anesthetized for microscopic analysis. Additionally, larvae were pretreated with different concentrations of Lip-Rutin before CDDP exposure to evaluate protective effects.

### Animals and CDDP ototoxicity model

A total of 20 male C57BL/6 mice, aged 6 weeks and weighing 18 to 20 g, were obtained from the Hubei Experimental Animal Research Center. After a 2-week acclimation period, mice were randomly allocated into 4 treatment groups (*n* = 5 per group): (a) control receiving daily intratympanic saline (6 μl); (b) Lip-Rutin receiving daily intratympanic Lip-Rutin (6 μl, 1 mM); (c) CDDP receiving daily intraperitoneal CDDP (3 mg/kg) with concurrent intratympanic saline (6 μl); and (d) combination receiving daily intraperitoneal CDDP (3 mg/kg) with concurrent intratympanic Lip-Rutin (6 μl, 1 mM)—all treatments administered for 7 consecutive days. All procedures adhered to NIH Guidelines and were approved by the Animal Care Committee of Tongji Medical College (IACUC Number: 3532).

### Assessing damage to hearing and HCs in mice

To investigate the in vivo distribution of liposomes, liposomes containing 1,1′-dioctadecyl-3,3,3′,3′-tetramethylindodicarbocyanine perchlorate (DID) and rutin were labelled as Lip-Rutin-DID. In vivo imaging of mice was performed at specified time points (2, 24, 48, 72, and 96 h) post-administration. Following euthanasia, cochleae were harvested for in vitro fluorescence imaging. Auditory brainstem response (ABR) tests assessed auditory thresholds at various time points post-injection [12]. Mice were anesthetized, electrodes were inserted, and auditory stimuli were presented to determine the ABR threshold. Cochleae were then collected and decalcified, and the basilar membrane was dissected for MYO7A staining and HC quantification using confocal microscopy. Following euthanasia, temporal bones were rapidly dissected on ice. The cochlear basilar membrane was micro-dissected using angled forceps, flash-frozen in liquid nitrogen, and homogenized in radioimmunoprecipitation assay (RIPA) buffer containing protease/phosphatase inhibitors. The extracted proteins were subsequently subjected to Western blot analysis to assess the expression levels of target proteins.

### Biocompatibility assay

After 14 days, mice treated with saline and Lip-Rutin were euthanized, and organs were excised for histological staining. Each mouse provided 0.3 ml of serum for analysis. Liver and kidney functions were evaluated using a biochemistry analyzer.

### Statistical analysis

All data are presented as the mean ± standard deviation (SD). Differences between 2 groups were assessed using Student’s *t* test, while differences among multiple groups were evaluated using 2-way analysis of variance. A significance threshold was set at *P* < 0.05.

## Results and Discussion

### Synthesis and characterization of Lip-Rutin

Liposomes were created using the thin-film hydration technique, employing specific ingredients in various ratios. The encapsulation efficiency (EE%) and particle size were influenced by the rutin/liposomes ratios. To optimize encapsulation efficiency, drug loading, and particle size, a ratio of 1:6 was selected to create the Lip-Rutin solution (Fig. S1A to C). In the optimized formulation, Lip-Rutin demonstrated 83.40% encapsulation efficiency and 13.90% drug loading, ensuring sufficient payload delivery for subsequent experiments.

**Fig. 2.**
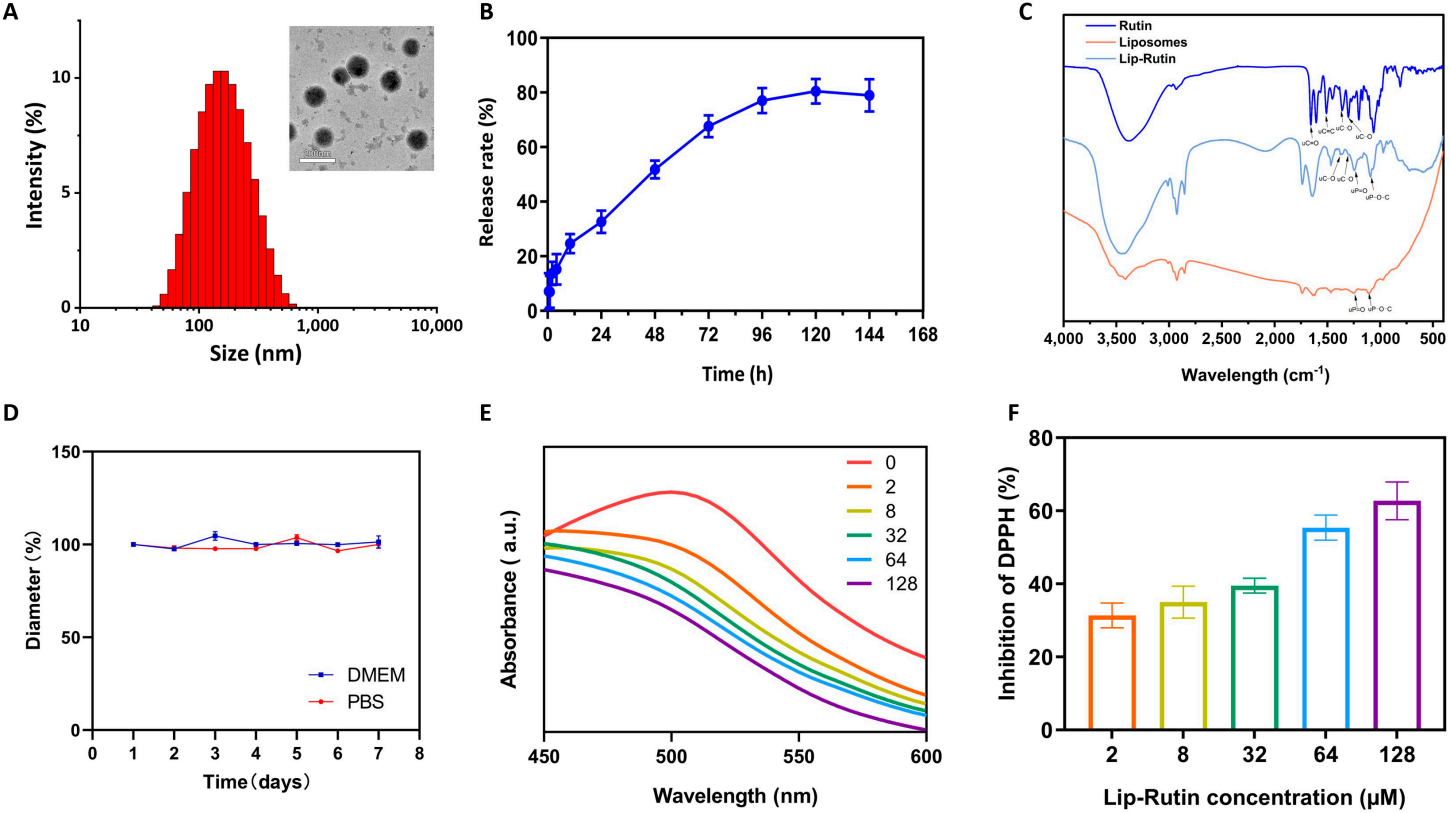
Synthesis and characterization of Lip-Rutin. (A) Particle size and representative TEM images of Lip-Rutin. Scale bar: 200 nm. (B) Rutin release from Lip-Rutin in vitro. (C) FTIR spectra of rutin, liposomes, and Lip-Rutin. (D) Stability of Lip-Rutin in high-glucose DMEM and PBS (*n* = 3). (E) The absorbance of DPPH with varying concentrations of Lip-Rutin. (F) The inhibition of DPPH by varying concentrations of Lip-Rutin at a wavelength of 517 nm (*n* = 3).

The prepared Lip-Rutin solution exhibited a light yellow, turbid appearance, whereas the rutin solution directly dissolved in DMSO appeared as a bright yellow liquid. The blank liposome solution had a milky white, turbid appearance (Fig. S1D). DLS analysis revealed that Lip-Rutin had a mean particle size of 144.6 nm, a PDI of 0.267, and a zeta potential of −9.27 mV (Fig. 2A). These characteristics may facilitate their entry into the cochlea and prolong their retention within the cochlea [29,30]. Electron microscopy confirmed that Lip-Rutin structures were smooth and spherical, consistently sized around 150 nm with a uniform distribution (Fig. 2A).

The release profile of Lip-Rutin was analyzed at room temperature. An initial rapid release of 24.6% of rutin occurred within the first 10 h, followed by a steady release from 24 to 72 h, ultimately releasing approximately 67.8% of the encapsulated rutin. The release rate then decreased, leading to an overall release of about 80.5% (Fig. 2B). FTIR results revealed the typical peaks of the C=O bond (υC=O) at 1,660 cm^−1^, aromatic C=C bond (υC=C) at 1,504 cm^−1^, phenolic C–O bond (υC–O) at 1,362 cm^−1^ and 1,297 cm^−1^ in rutin, and P=O bond (υP=O) at 1,240 cm^−1^ and P–O–C bond (υP–O–C) at 1,084 cm^−1^ in liposomes. Furthermore, the 1,362 and 1,297 cm^−1^ peaks of the phenolic group in rutin and the 1,240/1,084 cm^−1^ peaks of P=O/P–O–C in lipids were all present in Lip-Rutin, indicating the successful encapsulation of rutin in the liposome (Fig. 2C). Lip-Rutin maintained its particle size and PDI over a duration of 7 days in DMEM and PBS, reflecting its high degree of stability (Fig. 2D).

The antioxidant activity of Lip-Rutin is demonstrated through the scavenging of DPPH radicals [26]. As Lip-Rutin concentration increases, DPPH absorbance continuously decreases, indicating radical consumption (Fig. 2E). At 517 nm, Lip-Rutin exhibits scavenging activity even at low concentrations, with inhibition rates rising to 73.2% (Fig. 2F). These antioxidant properties are likely to mitigate CDDP-induced ototoxicity.

### Cellular uptake

**Fig. 3.**
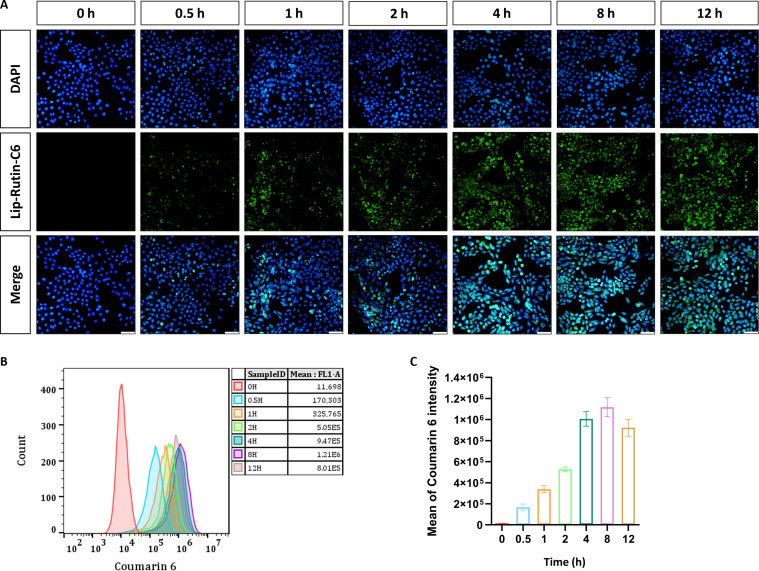
Cellular uptake characteristics. (A) Fluorescence microscopy images of OC-1 cells after incubation with Lip-C6 for various durations (0.5, 1, 2, 4, 8, and 12 h). Scale bar: 50 μm. (B) Flow cytometry assay. (C) Mean fluorescence intensity of OC-1 cells by flow cytometry assay (*n* = 3).

To substantiate the cellular uptake characteristics, Lip-Rutin-C6 was prepared by encapsulating coumarin-6 in liposomes for further investigation. Fluorescence microscopy revealed that Lip-Rutin-C6 predominantly accumulated within the cytoplasm of the cells, with the fluorescence intensity in OC-1 cells increasing over time and remaining high (Fig. 3A). This observation was corroborated by flow cytometry results, which showed sustained liposome uptake by OC-1 cells after 4 h (Fig. 3B and C). These findings suggest that employing liposomes as drug carriers is a stable and reliable approach for the effective delivery and release of rutin within OC-1 cells.

### Lip-Rutin mitigated CDDP-induced oxidative damage and mitochondrial dysfunction

To develop the CDDP-induced OC-1 cell toxicity model, OC-1 cells were exposed to varying concentrations of CDDP (2.5, 5, 10, 20, 40, and 80 μM) for 24 h. Based on CCK-8 assay results, cell viability decreased to approximately 50% at 10 μM CDDP, confirming this concentration as effective for inducing cellular damage (Fig. S2A). Lip-Rutin treatment at various concentrations did not induce cytotoxicity in OC-1 cells (Fig. S2B). However, pretreatment with Lip-Rutin mitigated cellular damage following CDDP exposure, with higher Lip-Rutin concentrations reducing CDDP-induced cytotoxicity (Fig. S2C). Consequently, a concentration of 50 μM Lip-Rutin was selected for the treatment group.

**Fig. 4.**
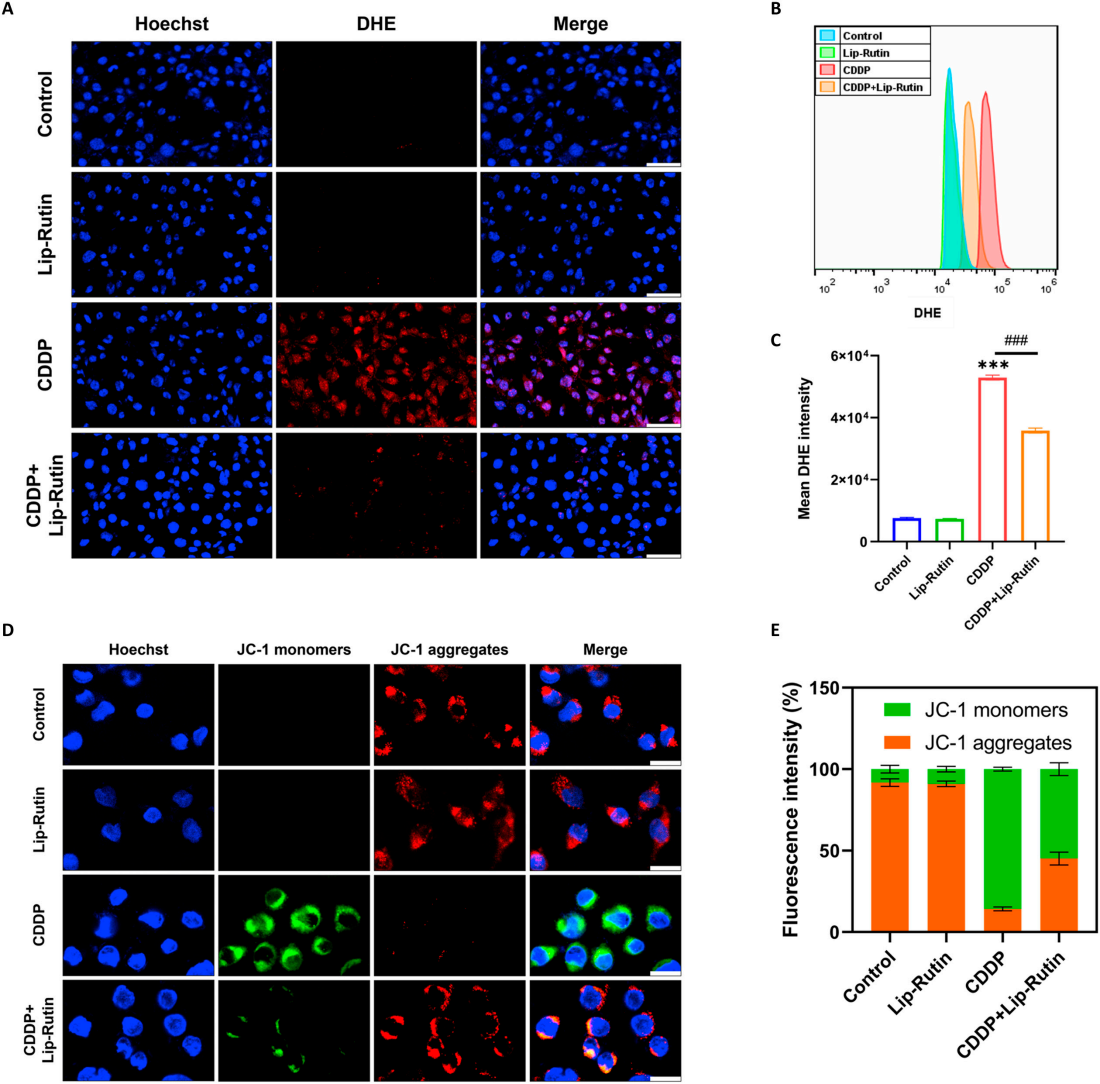
Detection of ROS levels and mitochondrial dysfunction. (A) Confocal images showing intracellular ROS stained by DHE in the control, Lip-Rutin, CDDP, and Lip-Rutin pretreated groups. Scale bar: 50 μm. (B) Flow cytometry analysis of DHE fluorescence intensity in different groups. (C) Quantification of DHE fluorescence intensity by flow cytometry (*n* = 3). (D) Representative fluorescence images of OC-1 cells stained with JC-1 (red represents JC-1 aggregates at high mitochondrial membrane potential, and green represents JC-1 monomers at low mitochondrial membrane potential). Scale bar: 20 μm. (E) The proportions of JC-1 monomers and JC-1 aggregates in each group (*n* = 3). ***P < 0.001 compared to the control group. ###*P* < 0.001 compared to the CDDP group.

Previous research has demonstrated that CDDP-induced HC apoptosis is associated with increased ROS levels [31]. To measure ROS levels in OC-1 cells post-CDDP exposure, DHE probes were utilized. The fluorescence intensity of DHE-stained cells significantly increased with CDDP-induced damage, indicating higher ROS generation (Fig. 4A to C). To investigate the role of mitochondria in CDDP-induced apoptosis in HEI-OC1 cells, changes in MMP were analyzed using JC-1. In the control group, JC-1 formed aggregates on the mitochondrial membrane of OC-1 cells, emitting strong red fluorescence. Post-CDDP treatment, the fluorescence shifted from red to green, indicating JC-1 monomer formation and mitochondrial depolarization. Pretreatment with Lip-Rutin resulted in increased red fluorescence, suggesting that Lip-Rutin mitigated CDDP-induced mitochondrial damage (Fig. 4D and E).

### Lip-Rutin may attenuate CDDP-induced OC-1 cells’ apoptosis via the p53 pathway

**Fig. 5.**
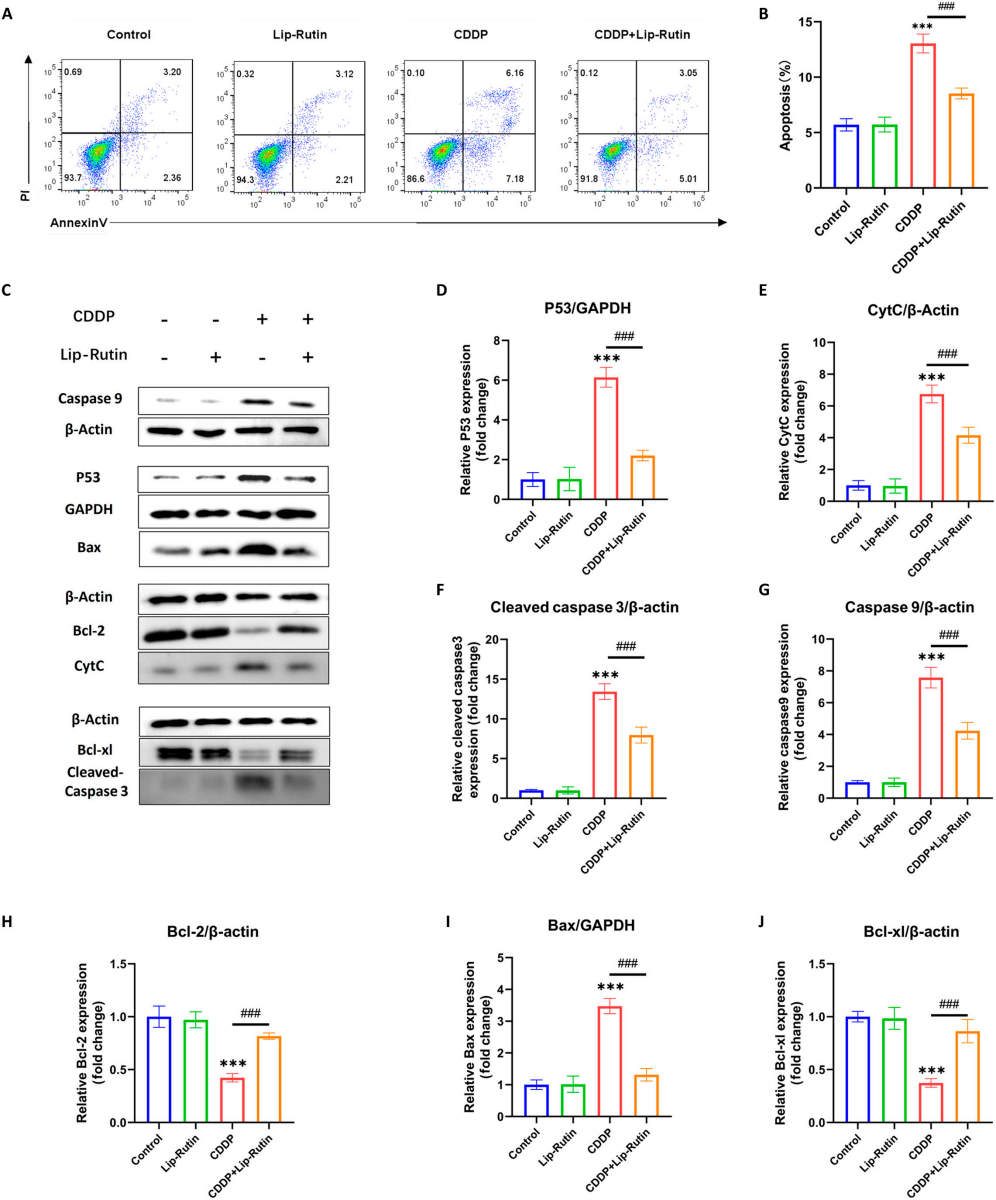
Effects of Lip-Rutin on CDDP-induced OC-1 apoptosis and Western blot analyses. (A) Flow cytometry assay of Annexin V/PI to assess apoptosis in OC-1 cells across different treatment groups. (B) Quantification of apoptotic cells from flow cytometry data (*n* = 3). (C to J) Representative Western blot images and relative expression of P53, CytC, cleaved caspase-3, caspase-9, Bcl-2, Bax, and Bcl-xl (*n* = 3). ***P < 0.001 compared to the control group. ###*P* < 0.001 compared to the CDDP group.

CDDP increased apoptosis rates in OC-1 cells, while pretreatment with Lip-Rutin mitigated this effect (Fig. 5A and B). Investigation into the underlying mechanisms revealed that CDDP administration up-regulated the P53 protein in OC-1 cells, altering the expression of BCL family proteins (Fig. 5C, D, H, I, and J). The expression of pro-apoptotic proteins CytC, caspase-9, and cleaved caspase-3 increased following these changes (Fig. 5C, E, F, and G). Compared to OC-1 cells treated solely with CDDP, the levels of pro-apoptotic proteins P53, Bax, CytC, caspase-9, and cleaved caspase-3 were significantly reduced in OC-1 cells pre-exposed to Lip-Rutin, while the levels of anti-apoptotic proteins Bcl-2 and Bcl-xl were notably elevated (Fig. 5C to J).

CDDP-induced activation of the P53 gene in OC-1 cells results in increased P53 protein expression and its translocation into the mitochondria [32,33]. This process activates Bax and inhibits Bcl-xl and Bcl-2, increasing mitochondrial membrane permeability, mitochondrial depolarization, and dysfunction [34]. These changes cause ROS accumulation in cells, up-regulate caspase-9 expression, and activate caspase-3 [35,36]. Concurrent mitochondrial changes trigger CytC activation and translocation, leading to caspase-9 activation and increased caspase-3 expression [37,38]. Caspase-3 is then cleaved to form cleaved caspase-3, inducing cellular apoptosis. Lip-Rutin efficiently eradicates ROS in cells and lowers P53 expression by inhibiting the P53 signaling pathway. This action suppresses the apoptotic process, safeguarding mitochondrial structure and function, thereby mitigating CDDP-induced damage to OC-1 cells. This conserved antioxidant efficacy aligns with rutin’s redox-modulating capacity reported by Wang et al. and Saafan et al. [39,40], further supporting Lip-Rutin’s therapeutic potential against CDDP-induced oxidative damage.

### Lip-Rutin reduces HC damage in mouse cochlear explants induced by CDDP

**Fig. 6.**
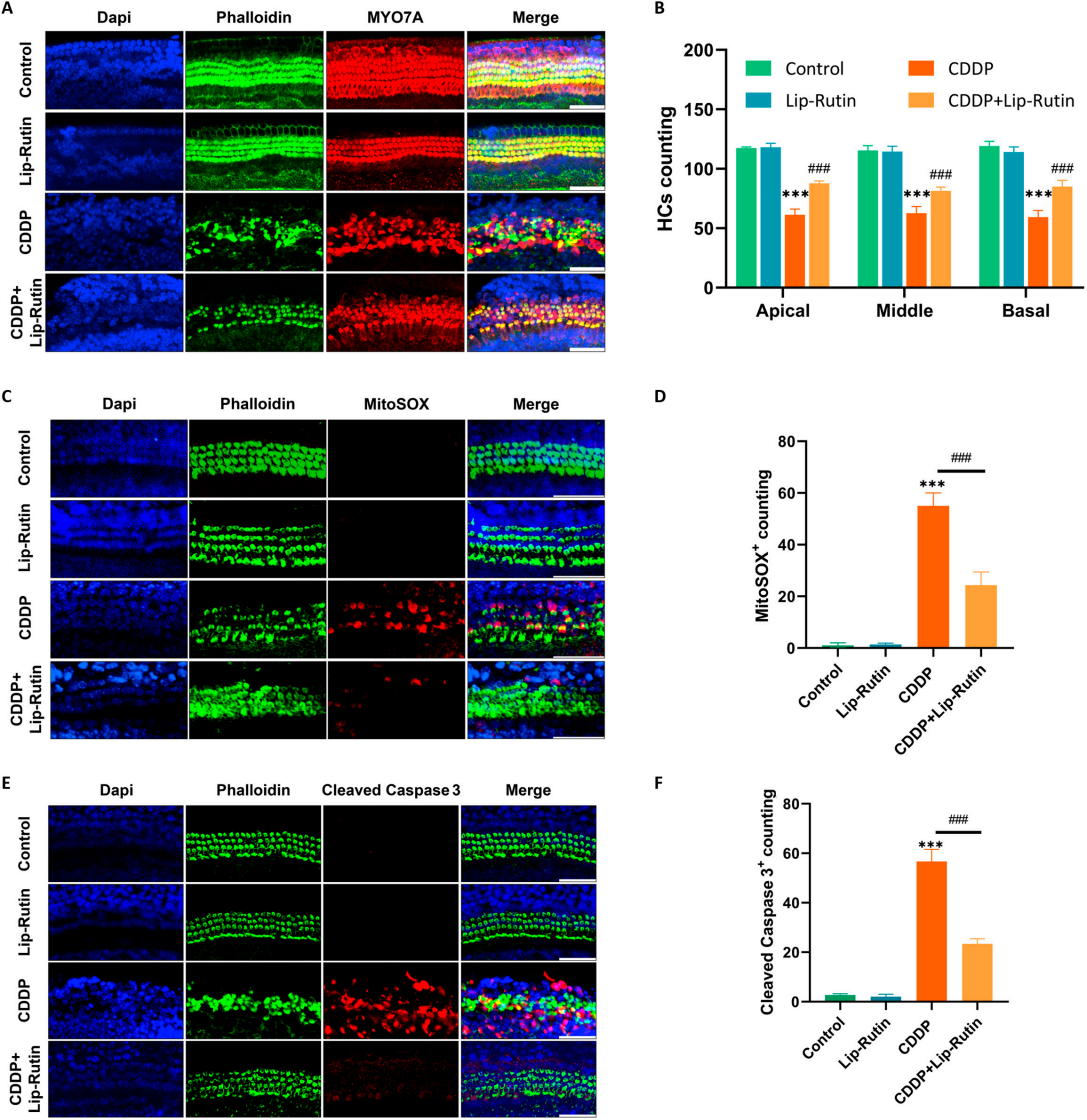
Lip-Rutin partially protected mouse cochlear explants from CDDP-induced damage. (A and B) Representative immunofluorescence images of HCs in the middle turns of the control, Lip-Rutin, CDDP, and Lip-Rutin pretreated groups, with the number of cochlear HCs counted every 200 μm (*n* = 4). Scale bar: 50 μm. (C) Representative images of HCs stained with MitoSOX (red) in the middle turns from different groups. Scale bar: 50 μm; (D) The number of MitoSOX-positive cochlear HCs counted every 200 μm. (E) Immunofluorescence staining of cleaved caspase-3 (red) in the middle turns of the cochleae from different groups. Scale bar: 50 μm. (F) The number of cleaved caspase-3-positive cochlear HCs counted every 200 μm. ***P < 0.001 compared to the control group. ###*P* < 0.001 compared to the CDDP group.

Following treatment with various concentrations of CDDP for 24 h, cochlear explants showed dose-dependent damage to HCs caused by CDDP. At a concentration of 12 μM, the survival of HCs was reduced, and there were observable changes in the morphology of HCs (Fig. S3A and B). Consequently, the treatment of 12 μM CDDP for 24 h was deemed as the most effective. Prior treatment of cochlear explants with Lip-Rutin (50 μM) before CDDP introduction significantly enhanced HC survival compared to those treated with CDDP alone (Fig. 6A and B). These results demonstrate the protective effects of Lip-Rutin on HCs in cochlear explants against CDDP-induced damage. Furthermore, the impact of Lip-Rutin on apoptosis in cultured cochlea was examined by staining for MitoSOX and cleaved caspase-3. The findings indicated a marked increase in MitoSOX-positive and cleaved caspase-3-positive cells in the CDDP-alone treated group. Conversely, a significant reduction in the ratio of MitoSOX and cleaved caspase-3-positive cells was observed in the Lip-Rutin pretreatment cohort compared to the CDDP-alone group (Fig. 6C to F). These observations suggest that pretreatment with Lip-Rutin inhibits the apoptotic cascade induced by CDDP.

### Lip-Rutin attenuates CDDP-induced hearing loss in vivo

**Fig. 7.**
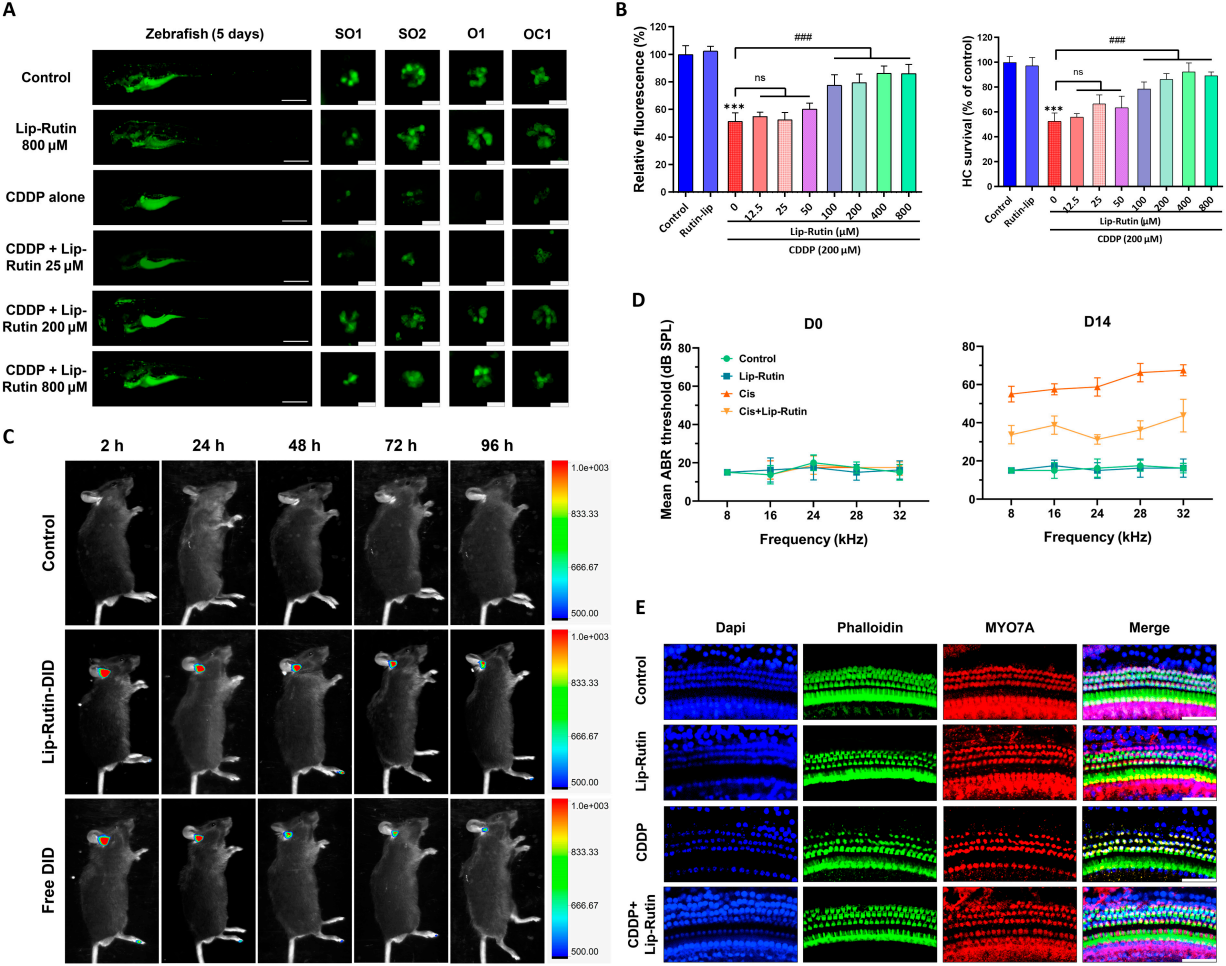
(A) Representative images of zebrafish larval stained by DASPEI in different groups. Zebrafish scale bar: 200 μm; neuromasts scale bar: 20 μm. (B) The relative fluorescence and HCs survival of zebrafish larva in different groups (*n* = 5). (C) In vivo fluorescence imaging of cochleae at indicated time points post-administration. (D) The ABR thresholds of mice with different treatments at different time points after CDDP injection. (E) Representative images of basilar membrane dissected from varying treated mice (*n* = 5). Scale bar: 20 μm. ***P < 0.001 compared with the control group. #*P* < 0.05; ##*P* < 0.01; ###*P* < 0.001 compared with the CDDP group.

To validate the protective effects of Lip-Rutin in vivo, its efficacy was tested on animal models. CDDP-induced damage in zebrafish larvae was evaluated by analyzing larval morphology, 2-[4-(dimethylamino)styryl]-1-ethylpyridinium iodide (DASPEI) fluorescence intensity, and HC survival [41,42]. Exposure to CDDP for 12 h caused carapace flexion and slight morphological changes at 100 μM. The fluorescence intensity of zebrafish larval DASPEI decreased with increasing CDDP concentration, showing a 50% reduction at about 192.3 μM. Similarly, the HC count exhibited a 50% damage level at around 214.6 μM (Fig. S4). To assess the protective efficacy of Lip-Rutin against CDDP-induced ototoxicity, we established an in vivo zebrafish model by exposing larvae to 200 μM CDDP for 12 h. Our findings demonstrate that pretreatment with 100 μM Lip-Rutin significantly attenuated CDDP-induced HC damage. This protective effect was evidenced by a significant enhancement in DASPEI fluorescence intensity, alongside the preservation of neuromast HC density and structural integrity (Fig. 7A and B). This protective effect aligns with Lip-Rutin’s established antioxidative properties observed in in vitro models, reinforcing its capacity to mitigate CDDP-induced oxidative injury.

In mice, CDDP treatment resulted in significantly elevated ABR thresholds across all frequencies, while mice treated with both Lip-Rutin and CDDP exhibited ABR thresholds significantly lower than those of mice treated with CDDP alone (Fig. 7D and Fig. S5). These results suggest that Lip-Rutin attenuates the elevation in ABR thresholds, thereby protecting against CDDP-induced hearing loss. To evaluate drug retention kinetics, we fluorescently labeled Lip-Rutin with DiD (Lip-Rutin-DiD) and monitored its cochlear distribution. Mice received intratympanic injections of saline, free DiD, or Lip-Rutin-DiD, followed by in vivo imaging at 2 to 96 h post-administration. Lip-Rutin-DiD demonstrated significantly stronger cochlear fluorescence intensity compared to free DiD, indicating prolonged inner ear retention (Fig. 7C and Fig. S6C). Ex vivo cochlear imaging confirmed enhanced Lip-Rutin-DiD penetration and sustained drug concentration within inner ear tissues (Fig. S6A and B). Immunofluorescence analysis revealed compromised morphology and a reduced HC count in CDDP-treated mice. In contrast, mice pretreated with Lip-Rutin displayed only slightly impaired HC morphology and significantly less HC loss compared to those treated with CDDP alone (Fig. 7E and Fig. S6D). Furthermore, cochlear analysis of Lip-Rutin-treated mice demonstrated significant down-regulation of p53 expression and concomitant reduction in caspase-9 activation and cleaved caspase-3 levels relative to CDDP-only controls (*P* < 0.01), mirroring the protective effects observed in our in vitro OC-1 cell model (Fig. S6E to H). This cross-model consistency strengthens the evidence for Lip-Rutin’s anti-apoptotic mechanism through p53 pathway modulation.

### In vivo biocompatibility evaluation

**Fig. 8.**
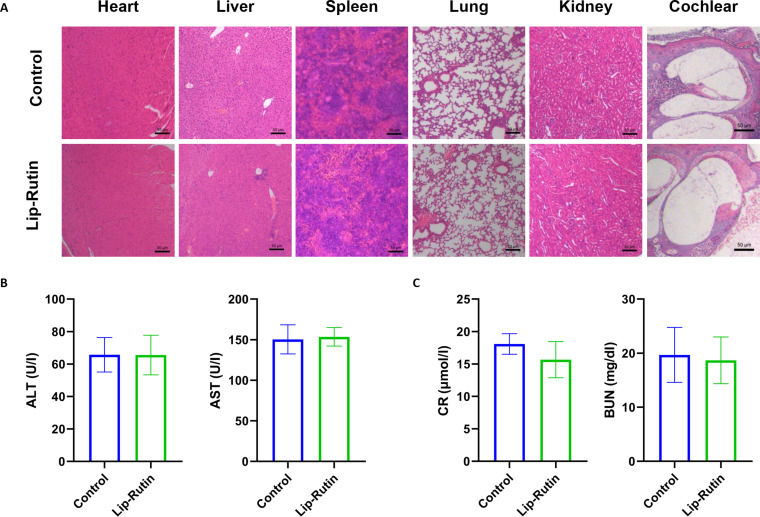
Safety evaluation in vivo. (A) Representative H&E staining of the major organs of mice treated with saline or Lip-Rutin. Scale bar: 50 μm. (B) Blood indices of ALT and AST for liver function assessment. (C) Blood indices of CR and BUN for kidney function assessment in mice with different treatments.

To evaluate the biosafety of Lip-Rutin, its in vivo toxicity was examined. Hematoxylin and eosin (H&E) staining of major organs (liver, heart, spleen, kidneys, and lungs) and cochlear tissues revealed no significant abnormalities in the Lip-Rutin treatment group compared to the saline group (Fig. 8A). Additionally, liver and kidney function parameters fell within normal reference values, indicating that Lip-Rutin did not induce noticeable hepatic or renal toxicity (Fig. 8B and C). These results collectively demonstrate that Lip-Rutin exhibits good biocompatibility and causes minimal toxicity to major organs and the inner ear.

## Conclusion

This investigation employed liposomes as nanocarriers to deliver the antioxidant rutin, aiming to prevent and mitigate CDDP-induced ototoxicity. The Lip-Rutin formulation developed in our research demonstrated smooth surfaces, optimal particle sizes, negative charges, excellent stability, and controlled release properties. Additionally, they exhibited potent radical scavenging capacity. Liposome-based drug delivery offers a promising therapeutic strategy for inner ear disorders by circumventing the BLB, enabling direct intracochlear drug delivery with enhanced inner ear concentrations while minimizing systemic side effects. Application of Lip-Rutin to OC-1 cells effectively reduced CDDP-induced oxidative stress and apoptosis. In cochlear explants, as well as zebrafish and mouse models, Lip-Rutin effectively alleviated CDDP-induced oxidative stress and apoptosis, offering substantial protection against CDDP-induced ototoxicity. These findings suggest that Lip-Rutin liposomal NPs hold promise as innovative therapeutic agents for preventing CDDP-induced ototoxicity.

## Data Availability

All data generated or analyzed during this study are included in this published article and the Supplementary Materials. The data that support the findings of this study are available on request from the corresponding author.
