# Supplementary material for: Liposome-Encapsulated Rutin Attenuates Cisplatin-Induced Ototoxicity via Suppression of P53-Associated Oxidative Injury
Source: Biomater Res. 2026 Feb 10;30:0324. doi: 10.34133/bmr.0324 (PMC12887162; doi:10.34133/bmr.0324)
Supplement: Supplementary 1 — Figs. S1 to S6 [file bmr.0324.f1.docx]

## Supportig works

- 1. **Synthesis and characterization of Lip-Rutin**


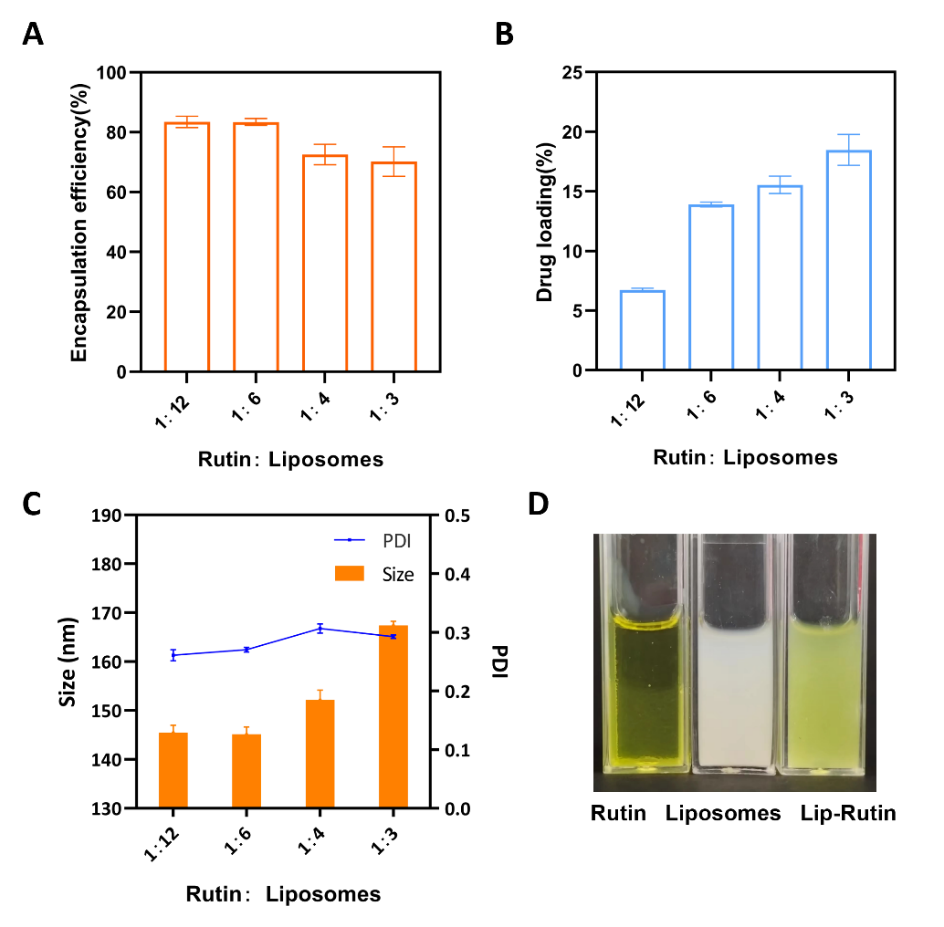


Fig. S1. Optimization and characterization of Lip-Rutin formulations at varying Rutin/Liposome ratios. (A) Encapsulation efficiency (%) at different Rutin/Liposome ratios; (B) Drug loading (%) at different Rutin/Liposome ratios; (C) Particle size and PDI at different Rutin/Liposome ratios; (D) Appearance of rutin solution, blank liposome solution, and Lip-Rutin solution.

- 1. ***In vitro* cytotoxicity**


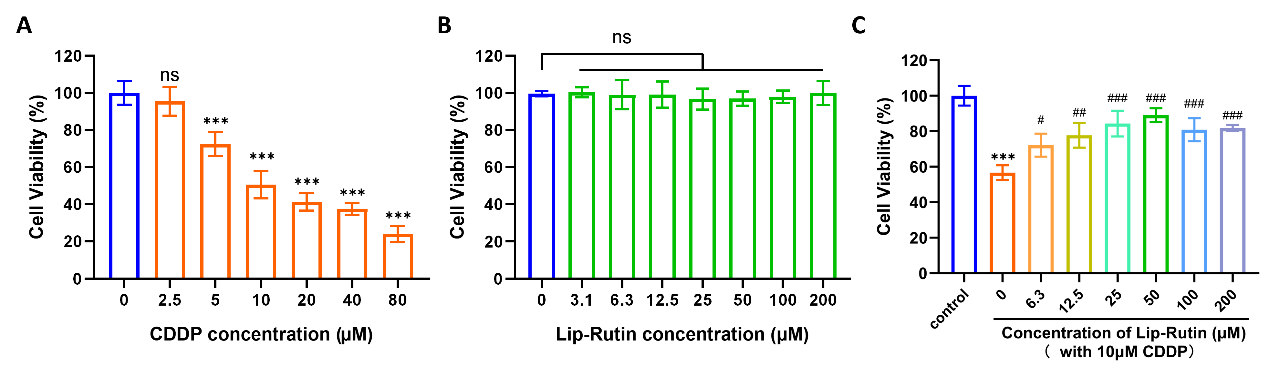


Fig. S2.  *In vitro* cytoprotective effects of Lip-Rutin against CDDP-induced ototoxicity in OC-1 cells. (A) Viability of OC-1 cells treated with various concentrations of CDDP. (B) Cell viability of OC-1 cells treated with various concentrations of Lip-Rutin. (C) Cell viability of OC-1 cells pretreatment with Lip-Rutin for 4 hours prior to CDDP exposure. ***p < 0.001; ns p≥0.05 compared with the control group. #p＜0.05；##p < 0.01; ###p < 0.001 compared with the CDDP grooup.

- 1. **Hair cells damage in mouse cochlear explants induced by CDDP.**


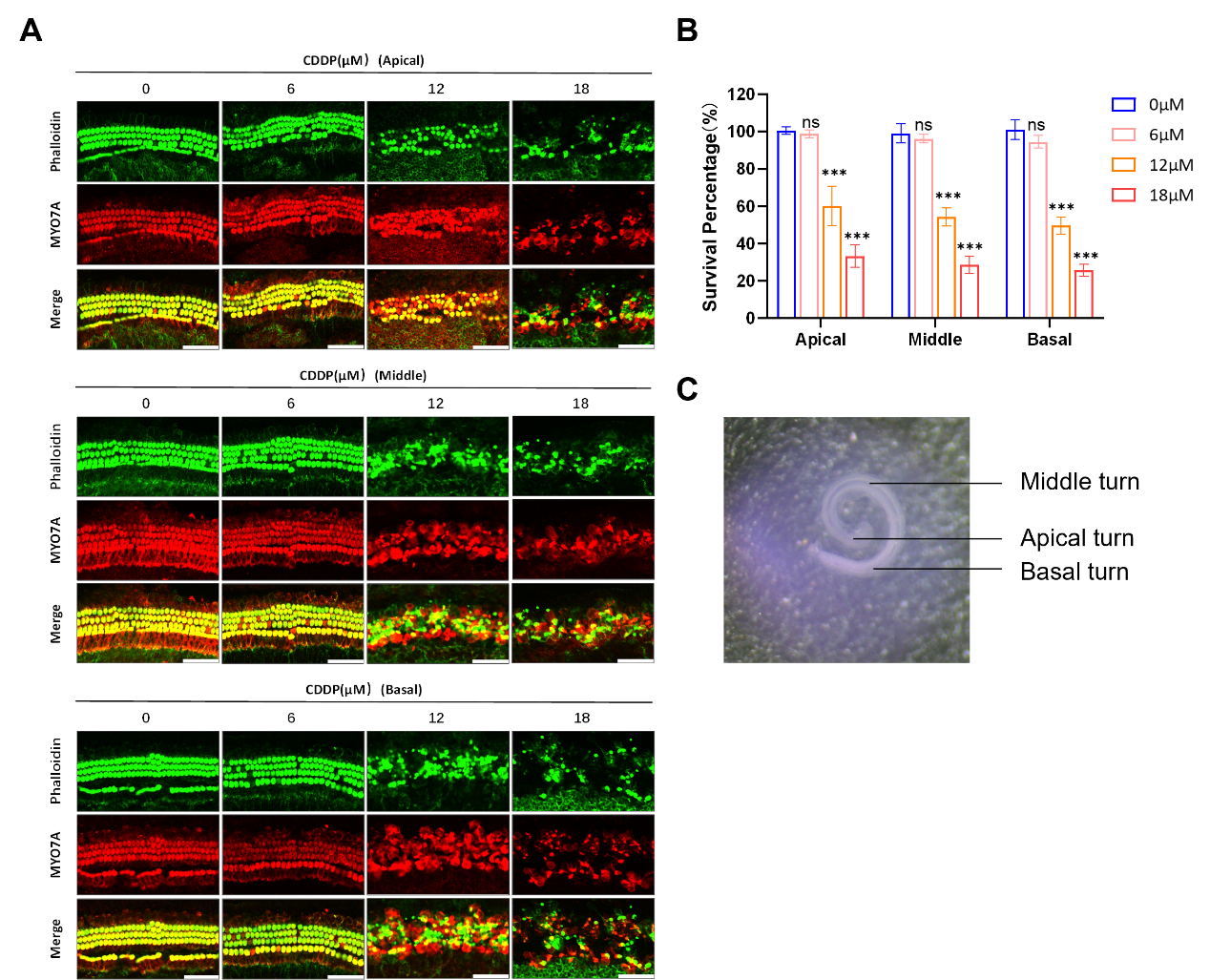


Fig. S3. Dose-dependent ototoxicity of CDDP in cochlear explants. (A) Representative images of HCs stained with myosin 7a and phalloidim in the apical, middle and basal turns treated with varying concentrations of CDDP. (Scale bar: 50 µm); (B) The survival percentage of cochlear HCs treated with CDDP in the apical, middle and basal turns; (C) The apical, middle, and basal turns of the cochlear explants.

- 1. **Hair cells in zebrafish from damage caused by CDDP.**


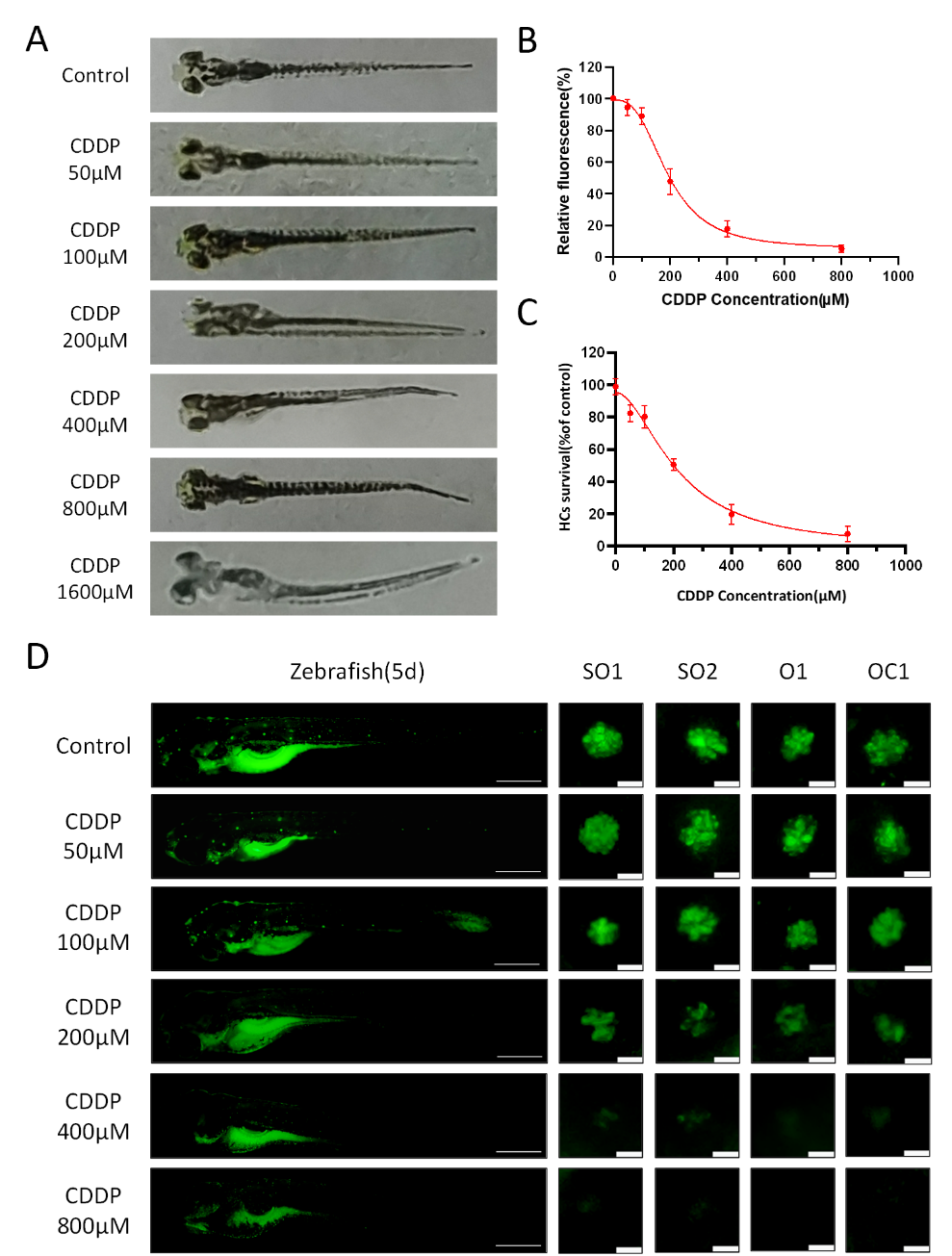


Fig. S4. CDDP-induced damage in zebrafish. (A) Morphology of zebrafish larval treated with varying concentrations of CDDP. (B) (C) The relative fluorescence and HC survival of zebrafish larval stained with DASPEI and treated with varying concentrations of CDDP treated. (D) Representative images of zebrafish larval stained with different concentrations of CDDP. (Zebrafish scale bar: 200 µm; neuromasts Scale bar: 20 µm)

- 1. **ABR** **thresholds of mice with different treatments**


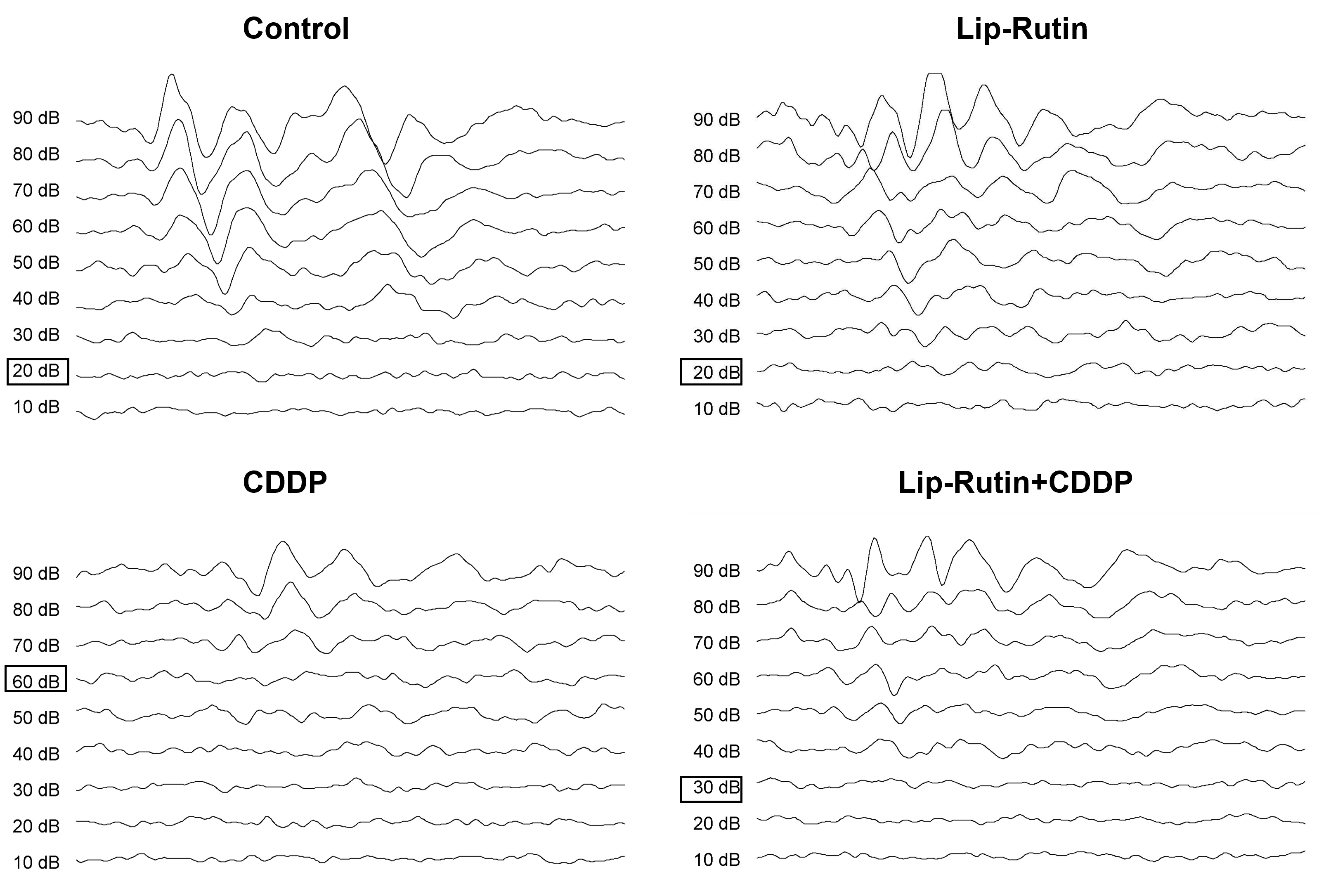
Fig.S5. ABR waveforms at 24 kHz on Day 14 post-CDDP exposure. Black boxes denote group-specific thresholds, revealing significant inter-group differences

- 1. **In vivo biodistribution and otoprotective efficacy of Lip-Rutin in mice.**


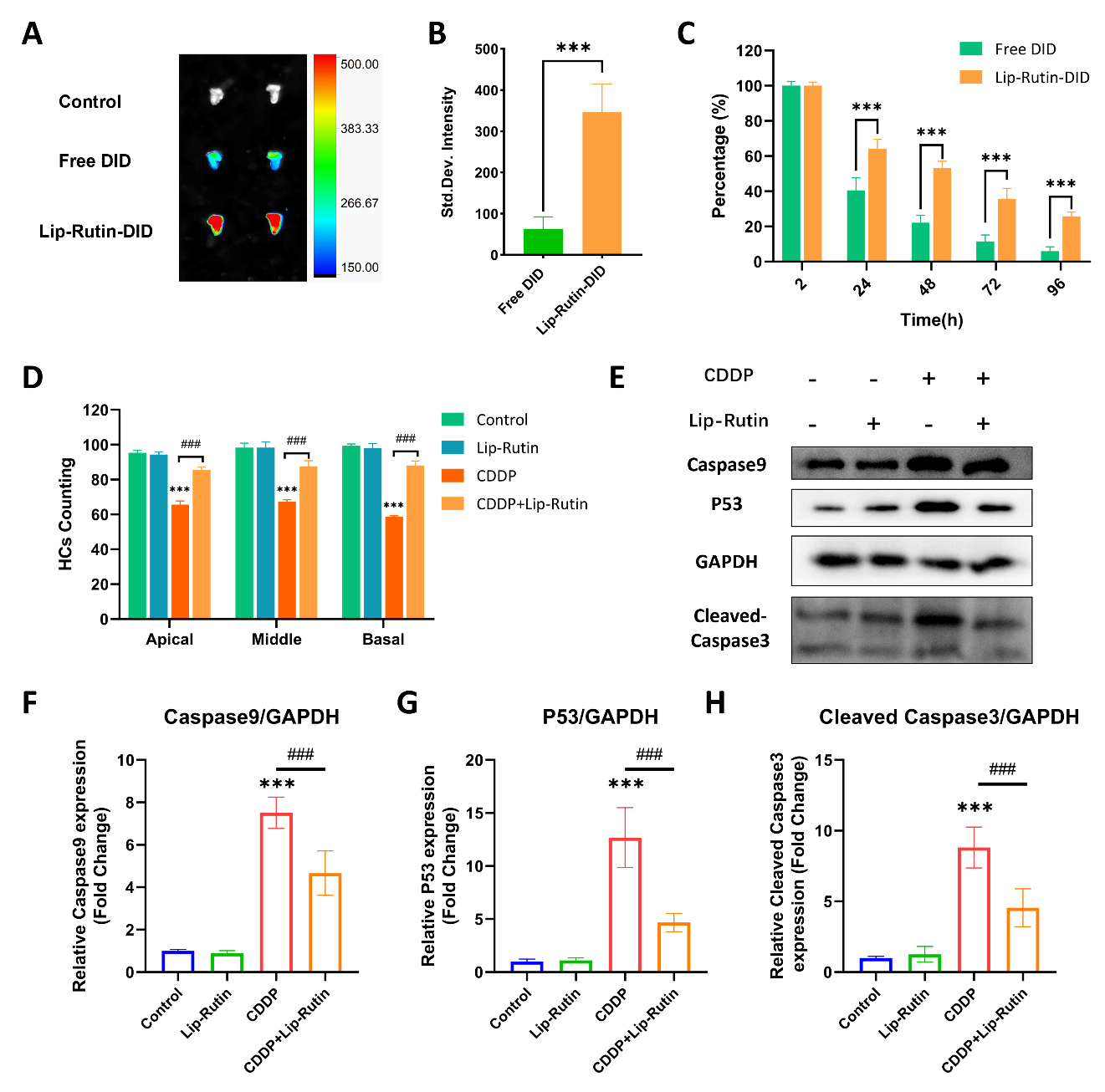


Fig.S6. Spatiotemporal distribution and otoprotective effects of Lip-Rutin. (A)(B) Ex vivo cochlear imaging (A) and quantitative fluorescence intensity (B) demonstrating Lip-Rutin accumulation in the cochlear. (C) *In vivo* fluorescence intensity of cochlear at indicated time points post-administration. (D) Quantification of hair cell survival per 200 μm of the cochlear basilar membrane, showing significant protection by Lip-Rutin. (E)~(H)Representative Western Blot images and relative expression of.
